# Supplementary material for: Are carbon emissions trading and green financial instruments synergistic? -Comprehensive quantitative research based on content analysis
Source: PLoS One. 2024 Mar 7;19(3):e0298601. doi: 10.1371/journal.pone.0298601 (PMC10919601; doi:10.1371/journal.pone.0298601)
Supplement: S1 Data — (ZIP) [file pone.0298601.s001.zip › Data/地级市Z_F工作报告.zip!/║■▒▒/╗╞╕╘╩╨/└┤╫╘╣1⁄2╓┌║┼í╛┬φ┐╦╩2╛▌═°í┐_2007.docx]

各位代表：

　　现在，我代表市人民政府，向大会作政府工作报告，请予审议，并请市政协各位委员和其他列席人员提出意见。　　一、过去五年工作的回顾　　市二届人大一次会议以来，在市委的正确领导下，市人民政府坚持以邓小平理论和“三个代表”重要思想为指导，认真贯彻落实科学发展观，团结带领全市人民，抢抓机遇，开拓进取，经济建设、政治建设、文化建设和社会建设都取得了丰硕成果。

　　(一)国民经济持续增长。地区生产总值由279.8亿元增加到380亿元，年均增长8.4%。三次产业比重由33.1:31:35.9调整为31.1:32.1:36.8。财政收入由21.8亿元增加到34.5亿元，年均增长9%。农业和农村经济稳步发展。粮食总产达到56.7亿斤，比2001年增产8亿斤。特色农业板块基地发展到625.8万亩，建成市级以上农业产业化龙头企业60家。累计培训农村劳动力150万人，转移129万人。工业经济发展速度与质效同步提升。规模以上工业增加值达到58.7亿元，年均增长16.8%。新增规模以上工业企业136家、产值过亿元的企业34家、年纳税过千万元的企业14家，创建湖北名牌产品19个、国家免检产品6个、省级出口品牌企业2家。现代服务业增长较快。第三产业增加值达到140 .2亿元，年均增长9.4%。全社会消费品零售总额184.5亿元，年均增长10.7%。 “万村千乡”工程顺利实施，城乡消费市场持续繁荣，金融、保险、旅游、通信和房地产等产业快速发展。

　　(二)发展后劲明显增强。固定资产投资年均增长15.6%，五年累计完成682亿元，开工建设重点项目106个。交通建设，境内国省主干线全部达到二级以上公路标准，通乡公路全面实现油路化，通村公路油路率达到42%。能源建设，完成了农网和城网改造工程，大别山火电厂、白莲河抽水蓄能电站建设进展顺利。农林水利建设，重点整险加固了长江干堤和20座大中型水库，长期困扰我市的防汛问题有效缓解;人工造林211万亩，退耕还林50.2万亩，森林覆盖率提高到43.8%。城镇建设，全面加强规划、建设和管理，城市化率达到36%。信息建设，实施了广播电视、电话“村村通工程”和“红色覆盖工程”。市区经济加快发展，规模以上工业增加值和财政收入跃升至全市第一位。县域经济和民营经济快速发展，县域规模以上工业增加值年均增长19.8%。个体私营企业发展到近10万户，民营经济增加值达到45亿元。

　　(三)改革开放深入推进。农村综合改革、国有农场管理体制改革以及粮食流通、农村卫生、血防等体制改革全面展开。落实农村“两免三补”政策，全市农民减负增收9.2亿元。企业改革不断深入，97.6%的国有企业实现了民营化。稳步推进财政体制改革，部门预算编制面达到100%，财政与编制政务公开范围扩大到609项资金和所有纳入编制管理的单位及其人员。推行“以票控费”、“联审联办”制度，行政服务中心建设进一步规范。投融资、流通、教育、文化和公务员管理体制改革取得新进展。土地收购储备和经营性土地“招拍挂”出让制度全面推行。国有资产运营管理得到加强。全市招商引资项目3800个，到位资金170亿元，其中亿元以上项目77个。新增外资企业158家，实际利用外资3亿美元，年均增长24.4%。一批国内外知名企业相继落户黄冈。外经外贸进一步扩大，累计出口7亿美元，年均增长15.4%，新增年出口额过百万美元的企业19家。积极开展了区域交流与合作，加快融入武汉城市圈和大京九经济协作带的进程。各类开发区较好地发挥了窗口、示范和辐射作用。

　　(四)人民生活不断改善。实施积极的就业政策，累计新增城镇就业岗位14.5万个，帮助下岗失业人员实现再就业13.5万人。着力完善社会保障体系，社会保险参保人数每年新增2万以上，五年4次调高离退休人员基本养老金、2次调高城镇低保标准。加大社会救助、救灾和扶贫工作力度，农村特困户救助对象18.1万人，农村贫困人口平均每年减少13万人。重视解决城乡困难群众的生产生活问题，多途径安排残疾人就业，农村“五保”老人集中供养率达到80%以上。城镇居民人均可支配收入、农民人均纯收入分别达到6981元、2861元，年均增长8.7%和6.6%。全市人均储蓄存款由1980元增加到4725元。城乡居民食品支出占消费总支出的比例(恩格尔系数)分别为36.6%和45.9%，五年下降了3.2个和2.5个百分点。居民消费价格涨幅连年控制在3%以内。切实维护群众的合法权益，清理建设领域拖欠工程款和农民工工资、医疗教育等领域“乱收费”治理、食品药品安全监督等专项工作取得明显成效。市政府每年承诺并办好8到10件实事，解决了一批事关群众切身利益的突出问题。

　　(五)社会事业长足进步。扎实推进“科技兴市”战略，实施科技项目500多项，省级以上高新技术企业发展到41家，科技进步贡献率达到45%。优先发展教育事业，全市预算内义务教育经费年均增长12%。适龄儿童入园率达到60.6%，小学入学率99.9%，初中入学率97.9%，高考上本科万人比居全省前列。省部级以上重点职业教育学校发展到17所，高等院校增加到4所。文化事业和文化产业加快发展，广泛开展群众文化活动，加强了文物和非物质文化遗产保护，推出了一批优秀文艺创作人才和精神产品。《黄冈市志》正式出版。卫生事业健康发展，三级医疗预防保健网逐步健全，新型农村合作医疗扩大到4个县市，试点县农民参合率达到82.5%。战胜了“非典”和禽流感疫情，艾滋病、血吸虫病等重大传染病和地方病防治取得新进展。群众性体育活动日趋活跃，竞技体育水平不断提高。高度重视人口、资源、环境工作，出生人口性别比偏高的问题得到有效遏制，土地、水、森林、矿产等资源保护力度加大，环境质量总体保持稳定。民族宗教、外事侨务、对台、人防、信访、气象、地震、档案、保密、妇女、儿童、老龄等各项工作都取得了新的成绩。

　　(六)民主法制得到加强。广泛开展群众性精神文明创建活动，文明社区、文明家庭、文明单位、文明村镇、文明行业等各项创建活动全面推进。深入贯彻依法治市方略，坚持依法行政，加强法制宣传教育，公民法制观念明显增强。积极开展“双拥”活动，军政军民关系进一步密切，国防动员和民兵预备役工作有新的进步。加强社会治安综合治理，深入开展“打黑除恶、反盗打抢、查禁黄赌毒”等专项行动，依法惩处犯罪分子， “平安黄冈”建设取得实效。认真落实安全生产责任制，加强安全生产基础工作，安全生产和消防工作连年受到省政府表彰。深入开展反腐败斗争，领导干部廉洁自律、大案要案查处和源头治腐工作力度加大，警示教育、经济审计、行政监察和舆论监督全面加强，廉政建设取得新成效。

　　刚刚过去的2006年，全市上下紧紧围绕年初确定的工作目标，团结拼搏，克难奋进，实现了“十一五”的良好开局。据统计，地区生产总值增长10%，规模以上工业增加值增长25.5%，全社会固定资产投资增长26.7%，财政收入增长18.3%，社会消费品零售总额增长14.6%，外贸出口增长31.1%，实际利用外资增长34%，城镇居民人均可支配收入和农民人均纯收入分别增长9.8%和8.2%，居民消费价格涨幅为2.2%，城镇登记失业率为4.12%，人口自然增长率为3.33‰。市二届人大五次会议确定的10件实事基本完成。

　　各位代表，过去的成绩来之不易。这是省委、省政府和市委正确领导，全市人民同心同德、艰苦奋斗的结果;是历任黄冈领导打下良好基础和市人大依法监督、市政协民主监督的结果;是驻黄部队、武警官兵，各民主党派、工商联、无党派人士、各人民团体和社会各界共同努力的结果;是国内外黄冈籍老乡、各界朋友和外来客商鼎力支持的结果。在此，我代表市人民政府，向全市各条战线的广大干部群众、向所有关心支持黄冈发展的海内外人士，致以崇高的敬意和衷心的感谢!

　　在肯定成绩的同时，我们也清醒地认识到，当前我市经济社会生活中还存在不少矛盾和问题：综合实力不强，产业结构不优，工业化、城市化水平不高;农业抗御自然灾害和市场风险的能力仍然脆弱，农民持续增收难度较大;各级财政财力不足，社会事业欠账较多，就业和社会保障压力较大，部分群众生活比较困难;发展环境仍需改善，政府自身改革和职能转变还不到位，政府工作落实不够。对此，我们必须高度重视，采取有力措施，逐步加以解决。

　　二、今后五年政府工作的指导思想和奋斗目标　　今后五年，是我市体制转轨、经济转型、增长方式转变的关键五年，是全市人均GDP突破1000美元、发展面临竞争更激烈和矛盾更突出的挑战期，更是经济社会大发展、和谐黄冈大建设的机遇期。世界范围产业转移加快，国际经济技术合作加深，为我们利用国际资本、技术、市场提供了更多的机会和更广阔的空间。中央加大对中西部地区、革命老区、贫困地区和新农村建设的支持力度，我们可以争取更多的政策、项目和资金;中部崛起战略全面实施，武汉城市圈建设积极推进，有利于我们进一步加快发展;高速公路、高速铁路、黄金水道和能源、通信等大规模国家投资项目相继开工，给我们带来新的发展机遇。只要我们弘扬老区精神，抢抓新机遇，增创新优势，开创新局面，黄冈一定能在新的起点上创造新的辉煌!

　　按照市第三次党代会的决策部署，今后五年政府工作的指导思想是：坚持以邓小平理论和“三个代表”重要思想为指导，以科学发展观统领经济社会发展全局，以改革、开放、调整、创新为动力，继续按照“全党抓经济，重点抓工业，突出抓招商，着力抓环境”的总体思路，在更高层次上实施“生态立市、工业强市、开放活市、特色富市、科教兴市”的发展战略，统筹推进经济建设、政治建设、文化建设、社会建设，加速构建实力黄冈、活力黄冈、魅力黄冈、文明黄冈、和谐黄冈。

　　今后五年，全市经济社会发展的奋斗目标是：

　　经济实力有新跨越。全市主要经济指标年均增幅达到两位数以上，高于前一个五年的增长速度，高于全省平均水平，固定资产投资、财政收入、规模以上工业增加值年均增长率保持15%以上，力争经济总量指标在全省排位前移。经济运行的质量、效益进一步提高，二三产业在三次产业中的比重、财政收入占GDP的比重、税收占财政收入的比重明显上升。工业经济主导地位更加突出。加速形成一批聚集效应明显、特色突出、竞争力强的产业集群和工业园区，力争食品饮料、医药化工、纺织服装、建筑建材等支柱产业增加值占第二产业增加值的比重达到85%，每个支柱产业年产值过100亿元。社会主义新农村建设全面推进。建设一批特色鲜明、规模较大的优质农产品基地，培育一批实力雄厚、覆盖面广、带动力强的龙头企业，开发一批市场占有率较高的名优特新农产品，走出一条农业增产增效、农民持续增收、农村更加和谐的路子。第三产业对经济结构调整和增长方式转变的推动作用明显增强，旅游、房地产、文化教育、金融、信息、商贸等服务业实现突破性发展。

　　生态环境有新改善。着力推进资源节约和高效利用，重点行业、企业的能源、原材料、水资源和土地集约节约水平明显提高，循环经济总量快速提升，单位生产总值能耗比2006年降低20%。环境质量整体提高，重点流域、重点区域和重点城镇环境质量明显改善，清洁生产模式广泛推行，主要污染物排放总量削减5.6%。生态保护和建设得到加强，主要湖泊、水库水质达到Ⅱ类标准，饮用水源得到有效保护，水土流失治理和生态修复面积逐年增加。森林覆盖率提高到45%以上，城市建成区绿化率达到40%以上。

　　改革开放有新突破。企业改革、农村综合改革和行政管理体制改革进一步深化，财政、投融资、流通体制改革全面推行，文化、卫生、城建、科技、教育等领域的改革进展加快。大力调整和完善所有制结构，民营经济成为市域经济主体，全民创业成为经济社会发展的主旋律。对外开放不断拓展，经济外向度进一步提高，引进战略投资、跨国投资、建立友城关系等对外经济技术文化交流合作取得实质性进展。外贸出口年均增长15%以上，实际利用外资年均增长12%以上，招商引资实际到位资金年均达到80亿元以上。经济社会发展环境进一步改善，环境竞争力、市场化程度、外贸依存度明显提升。

　　城乡面貌有新变化。基础设施、基础产业和基础工作全面加强，交通、水利、能源、通信建设步伐加快，区位交通优势不断增强。市区加快完成基础设施硬化、绿化、亮化和江、河、湖、山景观建设，努力实现新兴工业城、文化教育城、商贸旅游城和滨江园林城的建设目标。全面推动县市城区和小城镇建设，着力强化县城的产业优势、风格特征、人口聚集和带动能力，建设一批经济繁荣、布局合理、设施配套、功能健全、环境优美、富有特色的新型小城镇。城市化率达到40%以上。农村基本实现“五改五治五通”，农民生产生活条件和居住环境进一步改善。

　　人民生活有新提高。劳动就业、社会保障、教育收费、医疗卫生、文化服务、收入分配、基础设施、住房供应、食品安全、安全生产、社会治安等关系群众切身利益的问题得到有效解决。城镇居民人均可支配收入、农民人均纯收入年均增长8%和6%左右。家庭财产普遍增加，居住条件进一步改善，城镇人均住房面积达到35平方米，农户住宅基本实现楼房化。基本解决38万绝对贫困人口的温饱问题。确保人民群众有基本医疗保障，饮用水有安全保障，困难群体有基本生活保障。社会治安状况进一步好转，80%以上的县市达到平安创建标准，人民群众的安全感和满意度明显提高。

　　社会和谐有新进展。社会就业比较充分，社会保障体系比较健全。5年新增就业岗位18万个，城镇登记失业率控制在4.5%以内。城镇养老、失业、医疗、工伤、生育保险覆盖率达到90%以上，农村低保制度全面建立，新型农村合作医疗覆盖率达到100%。科技进步贡献率提高5个百分点。教育水平稳步提高，文化事业不断繁荣，民主法制和精神文明建设全面加强，城乡文明程度和公民道德素质进一步提高。

　　三、切实做好2007年各项工作

　　今年全市经济社会发展的主要预期目标是：生产总值增长10%以上，全社会固定资产投资增长20%以上，社会消费品零售总额增长12%以上，外贸出口增长15%以上，利用外资增长12%以上，地方一般预算收入增长15%以上，城镇居民人均可支配收入增长10%，农民人均纯收入增长6%;居民消费价格涨幅控制在3%以内，城镇登记失业率控制在4.5%以内，人口自然增长率控制在6‰ 以内，亿元生产总值生产安全事故死亡率控制在0.51以内;单位生产总值能耗下降4%，主要污染物排放总量减少2%。

　　(一)加速推进新型工业化

　　大力实施“五个一百”工程，加快培育有竞争力的产业和骨干企业。重点抓好食品饮料、医药化工、纺织服装、机械电子、建筑建材等产业的发展，在优化结构、提高产品质量的基础上，扩大产能，延伸产业链，壮大产业群，提高产业竞争力。突出重点，扶优扶强，培植100亿元产业、壮大100家重点企业、抓好100个重点项目、新增100家规模以上企业、联系100家民营企业，力争再有20家企业进入全省“三个三”工程。

　　大力发展高新技术产业。加大资金、技术、人才等资源整合力度，着力培育一批高新技术骨干企业、农产品加工重点企业和重大科技成果转化企业。加快高新区、开发区和工业园区建设，理顺体制，完善规划，夯实基础，使之成为改革开放的试验区、招商引资的示范区和优势产业的聚集区。

　　大力培育精品名牌。积极推动资本、技术等要素向优势企业和优势产品聚集，引导企业以科技为支撑、以质量为中心、以市场为导向、以规模效益为目标，建立健全质量管理体系，千方百计提高产品的持久开发能力，打造自主品牌，引进著名品牌，争取年内再有一批产品跻身省级名牌。

　　大力优化投资结构，加强项目建设。抢抓中央加大直接改善农村生产生活条件的投入、支持基础教育和公共卫生等社会事业发展的投入、促进中部地区崛起等机遇，突破性地抓好固定资产投资和重点项目建设。进一步加大项目筛选、对接和衔接力度，加强重点项目建设的组织协调和服务，争取开工一批、建设一批、投产一批、储备一批，切实把结构调整、产业发展、企业成长和品牌培育落实到具体项目上。全市实施重点建设项目104个，完成投资147亿元。确保完成工业项目投资57亿元。扎实推进林纸一体化等重大项目的前期工作，力争尽早开工建设。

　　(二)扎实推进社会主义新农村建设

　　着力加强农村基础设施建设。认真落实支农惠农政策，加大对“三农”的投入。实施病险水库和连江支堤除险加固、大中型泵站更新改造、大型灌区节水改造等重点水利工程，抓好土地整理和高产农田建设。集中投入，强化措施，加快农村公路建设和农村饮水安全工程建设。加快农村沼气建设，新建沼气池1万口以上。完善农业生物灾害应急反应机制，健全防灾减灾体系，努力减轻动植物疫病和灾害损失。

　　着力推进农业结构调整。围绕发展高产优质高效生态安全农业，加大优势板块基地建设力度，做大做强特色产业，积极申报板栗、药材等原产地保护。加快奶牛、生猪、家禽高标准养殖小区和优质水产带建设。积极推进集体林权制度改革，探索实施低产林改造工程，加大岗地开发利用力度。

　　着力发展农业产业化经营。坚持为农民服务的方向，落实对农业产业化龙头企业的税收、信贷、科技扶持政策，加大农业招商引资力度，鼓励引导各类资本投资农业领域，加快引进培植一批起点较高、规模较大、带动力较强的大龙头、大集团。

　　着力推进农业科技推广应用和农村劳动力转移培训。推进基层农技、农机服务改革，积极探索新品种、新技术、新机具进村入户的有效机制和办法，大力提升农业标准化、机械化、信息化水平。把劳务输出作为产业来抓，深入推进农村劳动力转移培训，切实提高农民素质，增强就业创业能力，做大做强黄冈劳务品牌，力争劳务经济对农民增收贡献率达到60%以上。

　　着力加快小城镇和村庄建设。抓好城镇和村庄建设规划，大力发展特色产业，努力增强城镇的经济实力。不断总结经验，推广典型，用城市建设的理念建设管理小城镇。实施生态家园建设，推动农户改厕、改厨、改圈，整治村容村貌，方便村民生产生活。

　　(三)加快发展现代服务业

　　突破性发展旅游业。大力发展以革命传统教育为主题的红色旅游、以大别山生态风光为主题的绿色旅游、以历史文化和名胜古迹为主题的古色旅游，加强旅游基础设施建设，打造大别山旅游品牌。启动国家优秀旅游城市创建活动。积极申报大别山(黄冈)国家地质公园。

　　积极发展房地产业。实施住房发展“十一五”规划，调整住房供应结构，优化住宅空间布局，加大危旧房改造力度，建立和完善城镇廉租住房制度，重点抓好普通商品房和经济适用房建设。加强房地产市场的调控与管理，促进房地产业健康发展。健全住房公积金制度，促进住房消费稳步增长。

　　积极发展教育文化事业。加强教育资源的开发和利用，全面推进素质教育，创新教学模式，提高教育质量，增创教育优势，打造以黄冈中学为龙头的教育品牌。举办黄冈教育论坛，组建具有黄冈特色的职业教育集团，全面提升职业教育的竞争力与综合实力。积极扶植文化市场主体，加快发展广电、体育、黄梅戏、出版、报业、娱乐等文化产业。

　　优化金融服务，扩大信贷投入。积极推进信用黄冈建设，优化金融生态环境，争创A级金融信用市。激励支持金融机构创新产品，优化结构，扩大对重点建设项目和有市场、有效益企业的信贷投入，切实缓解中小企业贷款难的问题。发展“三农”信贷业务，改进农户小额信用贷款管理，做好扶贫贴息贷款工作。实施利用开发性金融合作规划，加强合作平台、融资平台、担保平台建设。建立完善信贷管理机制、贷款偿还机制和信用担保机制，促进金融业加快发展。

　　改造提升传统服务业。加快农村生产生活资料、农产品购销等流通服务网络建设，继续做好“万村千乡”试点工作，新建或改造一批标准化农家店。加强邮政服务“三农”站点建设，拓展农村商品连锁配送业务。鼓励支持中介、咨询和社区服务业。

　　(四)促进区域经济协调发展

　　进一步高昂市区龙头。注重从体制、机制上调动市、区两级发展的积极性，确保市区主要经济指标增幅高于全市平均水平。重点培育主导产业，加快工业园区建设，着力推进稳健二期、太子奶二期工程和伊利牛奶、乾龙精机等一批重点项目建设。坚持高起点规划、高标准建设、高效能管理，确保当年投入4.5亿元，实施18个重点城市建设项目。积极争创省级园林城市和国家级“双拥模范城”。

　　大力发展县域经济。认真贯彻“一主三化”方针，狠抓招商引资，发挥比较优势，壮大主导产业，优化发展环境。每个县市办好一个开发区或工业园区，发展2—3个支柱产业。鼓励支持基础较好的县　市率先建成经济强县，全面提升在全省县域经济中的位次。

　　加快融入武汉城市圈。重点围绕武汉现代制造业、高新技术产业、农副产品加工业以及都市旅游业等优势产业开展协作配套，努力使我市成为武汉制造业转移的重要吸纳地、特色农产品配送地和休闲旅游目的地。

　　(五)加强资源节约和生态环境保护

　　坚决落实节能减排任务。把节能减排作为考核企业的硬指标，作为考核各级领导的重要内容，严格落实节能减排责任制，层层分解目标任务，确保工作责任到位、政策落实到位、监督考核到位。切实加强用能大户的节能降耗工作，强力推进建筑节能，发展节能省地型建筑。落实燃煤大户的脱硫措施，加快污水处理设施建设。

　　大力发展循环经济。在建材、化工、冶金等资源消耗或废物产生量较大、污染较重的行业，实施减量化、资源化、再利用工程项目，建设一批循环经济示范园区和示范企业。大力培育循环经济产业链，推广生态农业模式。

　　淘汰落后生产能力。严格市场准入，坚决依法关闭不符合国家产业政策、严重浪费资源和污染环境的企业，加快淘汰落后的高耗能、高耗水、高耗材的工艺、设备和产品。认真实施小水泥专项治理工程。

　　加大环境和生态保护力度。坚决落实主要污染物总量控制目标，严格环保执法，严肃处罚违法行为。完善环境监测预警体系与环境监察体系，建立健全突发性重大环境事件预防与应急处理机制。进一步整顿和规范矿产资源开发秩序，抓好一批重点生态工程建设。

　　切实加强土地管理。严格执行国家土地调控政策措施，落实耕地保护行政首长负责制，坚决制止违法违规用地行为，抓紧完善和严格执行节约集约用地标准，提高土地利用效率。高度垄断土地一级市场，规范放开土地二级市场，加强出让国有土地使用权管理，提高土地资产效益。

　　(六)深入推进改革开放和自主创新

　　进一步深化各项改革。继续深化农村综合改革，着力抓好乡镇事业单位改革、农村义务教育体制改革和县乡财政体制改革。深化企业改革，引导企业建立规范的现代企业制度。国有商贸企业改制面达90%以上，部门企业改制面达80%以上。大力发展民营经济，建立健全领导和职能部门帮扶民营企业的长效机制。坚持勤俭节约，狠抓增收节支，加强税收征管，严厉打击偷骗逃税等违法行为。完善非税收入管理制度，硬化财政预算约束。改革公务活动接待制度，严格差旅费和会议费管理。深化行政管理体制改革，扩大财政、编制等政务公开的内容和范围。加快事业单位改革，重点推进全员聘用制。健全和完善国有资产监督管理体制，提高监管水平。

　　扩大对外开放。坚持不懈地抓好招商引资工作，突出招商重点，转变招商方式，提高招商引资质量，着力引进一批大项目、环保型项目、产业集群型项目。积极扩大外贸出口，重点抓好出口品牌和基地建设，支持高新技术产品、机电产品和板栗、茶叶、蜂蜜、水产品等农产品出口，提高纺织服装、生物医药出口产品质量、规模和档次。支持有条件的企业“走出去”，开展对外工程承包、服务外包和劳务输出。

　　加强自主创新能力建设。加强与科研院所和大专院校的联系与协作，完善创新激励机制，引导企业加大科技投入，加快科技创新和科技成果转化，为经济结构调整和增长方式转变提供科技支撑。

　　优化经济社会发展环境。进一步加强行政服务中心建设，规范行政审批事项，减少收费项目，简化办事程序，推进管理服务创新。建立首查先行责令改正制度、同城一家执法单位检查制度、检查评比处罚审批制度，完善办事公开承诺服务制、咨询首问负责制、重点项目专班负责制。加大经济社会发展环境治理力度，突出解决乱收乱罚、效率低下的问题，坚决打击“四强”、“四霸”。

　　(七)着力解决民生问题

　　积极扩大就业。强化政府促进就业职能，统筹做好城镇新增劳动力就业、农村富余劳动力转移就业、下岗失业人员再就业工作，力争城镇新增就业3.5万人。加大公益性岗位的开发力度，重点帮助“零就业”家庭和就业困难人员就业。积极促进残疾人、失地农民等特殊群体就业。加强劳动保护，维护农民工合法权益。

　　切实解决上学难、上学贵的问题。从今年春季开始，全部免除农村义务教育阶段学生学杂费，对贫困家庭学生免费提供教科书并补助寄宿生生活费。严格执行义务教育“一费制”收费政策和公办普通高中招收择校生“三限”政策，稳定高等学校收费标准，实行收费公示制。

　　逐步解决群众看病难、看病贵的问题。加快乡镇卫生院和村卫生室的改造和建设，推进新型农村合作医疗。加快构建以社区为基础的城市医疗卫生服务体系，在惠民医院开展医药分开新机制试点工作。整顿规范医疗服务和药品生产流通秩序，加强价格监管，降低医药费用。加强重大疾病预防工作，认真落实血吸虫病、艾滋病、结核病等重大传染病防治措施。高度重视和抓好防控人感染高致病性禽流感等工作。

　　完善社会保障和社会救助体系。进一步完善社会保险制度，逐步做实个人帐户，确保离退休养老金按时足额发放。加大社会保险扩面征缴力度，切实提高各类所有制企业的参保率，统一城镇个体工商户和灵活就业人员的参保办法，扩大社会保险的覆盖范围。支持发展面向“三农”的商业保险。加强城乡特殊困难群众社会救助体系建设，完善城镇低保、农村“五保”、农村特困户救助、大病医疗救助、灾民救助等制度。做好法律服务和法律援助工作，为困难群众打官司提供有效帮助。

　　加大扶贫开发力度。把扶贫开发与新农村建设、县域经济发展、现代农业建设紧密结合起来，突出抓好产业化扶贫、科技扶贫、招商扶贫、项目扶贫、搬迁扶贫和社会扶贫，在巩固整村推进的基础上，加快向整乡推进、区域推进发展。力争全市再减少绝对贫困人口7万人、低收入贫困人口12万人。

　　坚持不懈为群众办实事。今年市政府承诺办好8件实事：1、继续改善农民生产生活条件。硬化农村公路2000公里;解决23万农民的饮水安全问题;落实大中型水库移民后期扶持政策;加快农村特困户救助向农村低保过渡;为所有乡镇和80%的行政村提供互联网基础接入。2、做好就业培训转移工作。全市完成劳动力职业技能培训30万人次，培训转移农村劳动力10万人;为199个重点贫困村和老区重点村1000名特困家庭子女提供免费职业教育;资助8000名贫困村困难家庭子女进行专业技术培训，并确保转移就业。3、新增3个新型农村合作医疗试点县市，参合率确保80%以上;在黄冈市区开办一所惠民医院，办好社区卫生服务试点。4、将市直未参保的国有企业全部纳入基本养老保险范围，基本解决市直国有困难企业退休人员参加医疗保险问题。5、实施市级行政中心东移工程和遗爱湖治理保护工程，“刷黑”市区9条主要道路，完成20条背街小巷改造任务。6、对100所农村寄宿制中小学实施改水、改电、改厕、建沼气和改善住宿条件。7、实施城镇廉租住房制度，对符合条件的最低收入家庭落实住房补贴。8、新建黄冈市儿童福利中心。

　　(八)统筹经济和社会发展

　　加强社会主义精神文明建设。坚持科学理论指导，树立共同理想，弘扬民族精神、时代精神和老区精神，树立社会主义荣辱观，加强社会公德、职业道德、家庭美德教育，加强青少年思想道德建设，深入开展群众性精神文明创建活动。

　　加强社会管理，维护社会稳定。深入推进平安黄冈建设，加强社会治安综合治理，依法打击各种犯罪，增强人民群众的安全感。建立健全突发公共事件应急管理机制，有效预防和应对突发公共事件。加强食品、药品和餐饮市场监管，严厉打击制售假冒伪劣商品的行为。充分发挥价格杠杆作用，坚决治理乱收费、乱涨价。建立政府依法行政和社区依法自治的运行机制，加大对社区基础设施建设的投入，积极创建省级示范社区和文明社区。做好人民来信来访工作，畅通信访渠道，积极化解社会矛盾。

　　加强安全生产工作。深入开展安全生产落实年活动，强化安全生产责任制，严把安全准入关，加大安全生产监管和执法力度，坚决遏制重特大安全事故发生。

　　积极支持部队和国防建设。关心和支持驻黄解放军和武警部队建设，深入开展国防教育和双拥共建活动，健全国防动员体系，抓好人民防空和民兵预备役工作。积极做好部队转业干部、复员退伍军人接收安置、优抚和政策落实工作。

　　认真做好人口和计划生育、文化、体育等各项工作。进一步加强人口和计划生育工作，稳定低生育水平，深入开展“关爱女孩行动”，继续治理出生人口性别比偏高的问题。加强公共文化设施尤其是城乡基层文化设施建设，广泛开展群众性体育活动，提高竞技运动水平，办好第六届黄梅戏艺术节和第二届市运会。进一步繁荣新闻出版、广播影视等事业。加强统计工作，搞好农业普查。认真落实党的民族政策，依法管理宗教事务。做好外事侨务工作，扩大对外交往与合作。高度重视老龄工作，健全社会养老保障服务体系。关心支持妇女、儿童事业发展。认真做好对台、档案、地方志、保密等各项工作。

　　四、进一步加强政府自身建设

　　按照“为民、务实、清廉”的要求，全面加强政府系统思想、组织、作风和制度建设，不断提高政府执行力和公信力，树立法治政府、服务政府、责任政府、效能政府形象，全心全意为人民服务，对人民负责，受人民监督，让人民满意。

　　坚持科学行政。进一步转变政府职能，切实把政府抓经济工作的主要精力放在培育市场主体和创造良好的发展环境上。注重全面履行政府职能，更加重视社会管理和公共服务。按照精简、统一、效能的原则和决策、执行、监督相协调的要求，加强行政效能管理，消除梗阻，确保政令畅通。进一步完善政府工作目标管理责任制，加快建立以行政首长为重点的行政问责制度和绩效评估体系，在依法、依规、不留后遗症的前提下快干事、干实事、干成事。

　　推进民主行政。坚持市委统一领导，自觉接受人大及其常委会的依法监督，接受人民政协的民主监督，不断提高办理人大代表议案、建议和政协委员提案质量。认真听取各民主党派、工商联、无党派人士和工会、共青团、妇联等人民团体的意见，主动接受新闻舆论和社会公众监督，不断改进政府工作。提高行政管理透明度和公众参与度，健全重大决策的规则和程序，完善人民群众建议征集制度、重大事项社会公示制度、社会听证制度、专家咨询制度，完善政府信息公布制度，健全市长信箱、市长热线等政府与公众沟通机制。加强电子政务建设，高标准建设政府门户网站，促进政务公开，方便群众办事。

　　严格依法行政。全面实施依法行政纲要，加强政府法制工作，落实行政执法责任制，完善行政执法程序和行政复议制度，按照法定权限和程序行使权力、履行职责。开展“五五”法制宣传教育，增强公民尤其是各级领导干部的法律意识和法制观念。切实加强公务员法制教育和队伍建设，不断提高依法行政的素质和水平。

　　恪守廉洁行政。加强政府系统廉政建设，健全教育、制度、监督并重的惩治和预防腐败体系，形成靠制度管人管事制权的有效机制。严格执行土地批租、工程招投标、政府采购、产权交易等公开、公正、公平的各项规定，防止国有资产流失。强化监察、审计职能，加大内部监督力度。严明政纪，端正政风，切实解决以权谋私、与民争利和损害群众利益的问题。对违纪违法案件和腐败分子，依纪依法严肃惩处，决不姑息。

　　高效务实行政。建立高效协调的行政工作运行机制和求真务实抓落实的行政效能考核机制，大力精简会议、文件和各种检查评比，强化以办事效率、服务质量和工作纪律为主要内容的行政效能监察，坚持重实际，说实话，办实事，求实效，确保政府工作一件一件抓落实。

　　各位代表!新的征程任重道远，新的使命责无旁贷。让我们紧密团结在以胡锦涛同志为总书记的党中央周围，高举邓小平理论和“三个代表”重要思想伟大旗帜，全面贯彻落实科学发展观，在市委的正确领导下，紧紧依靠全市人民，万众一心，励精图治，坚韧不拔，继往开来，为建设富裕文明和谐的新黄冈而努力奋斗!
